# Unruptured cerebral aneurysms in elderly patients: key challenges and management

**DOI:** 10.1080/07853890.2021.1990393

**Published:** 2021-10-19

**Authors:** Nicholas Caffes, Nicole Wenger, Gregory Cannarsa, Jeffrey Oliver, Chimdiya Onwukwe, Dheeraj Gandhi, J. Marc Simard

**Affiliations:** a Department of Neurosurgery, University of Maryland School of Medicine, Baltimore, MD, USA; b Department of Radiology, University of Maryland School of Medicine, Baltimore, MD, USA; c Department Pathology, University of Maryland School of Medicine, Baltimore, MD, USA; d Department of Physiology, University of Maryland School of Medicine, Baltimore, MD, USA

**Keywords:** Elderly, aneurysms, unruptured, microsurgery, endovascular, challenges

## Abstract

Unruptured cerebral aneurysms are increasingly identified in elderly patients as the global life expectancy continues to rise and non-invasive vascular imaging becomes more prevalent. The optimal management of unruptured aneurysms in elderly patients remains controversial. Variability in life expectancy, comorbidities and rupture risk coupled with heterogenous endovascular and surgical treatments contribute to a paucity of clear guidelines, and current management is highly individualized. Elderly patients present unique considerations including frailty, cognitive dysfunction, vasculopathy, reduced life expectancy and overall worse prognosis in case of rupture which shape the risks and likelihood of success of endovascular and microsurgical treatment. In this review, we provide a comprehensive overview of unruptured cerebral aneurysms in the elderly, with a particular focus on the natural history, key challenges associated with advanced age, management and future innovations to further refine treatment.

**Key Messages**
1. The management of unruptured cerebral aneurysms in elderly patients remains controversial.
2. Key challenges including frailty, cognitive dysfunction, reduced life expectancy, vasculopathy and poor prognosis with aneurysm rupture add complexity to endovascular and surgical decision making not encountered with younger demographics.
3. A thorough understanding of available treatment options, likelihood of treatment success and associated risks weighed against the risk of aneurysm rupture informs patient discussion and management.

## Introduction

Life expectancy is increasing at unprecedented rates. By 2050, the proportion of individuals 65 years or older will more than double from 8.1% to 21% [1]. An ageing population presents unique challenges to the medical establishment, including neurological surgery. Increased availability and utilization of non-invasive neuroimaging is increasing the detection of unruptured cerebral aneurysms, management of which remains controversial in elderly patients.

Multiple guidelines regarding the management of unruptured cerebral aneurysms in the general population have been established by cornerstone neurosurgical trials [2]. Optimal management in the elderly, however, remains undefined. Incomplete and conflicting information regarding natural history coupled with increased prevalence of comorbidities, treatment risk and overall reduced life expectancy adds complexity not encountered in younger patients. Current practice adopts an individualized approach weighing the risk of aneurysm rupture against patient-specific comorbidities, life expectancy, treatment-related risks and likelihood of treatment success. This review provides a comprehensive overview of unruptured cerebral aneurysms in the elderly, with particular focus on key challenges influencing both endovascular and surgical decision making and outcomes. A precise understanding of rupture risk, recognition of demographic-specific challenges, thorough understanding of available treatment options and outcomes, as well as a cursory understanding of investigative treatments allows for informed discussion and management.

## Natural history

### Prevalence

The prevalence of unruptured cerebral aneurysms in the elderly and the association between age and aneurysm development remain controversial. Several autopsy reports describe an increased prevalence of unruptured cerebral aneurysms with advancing age. Inagawa et al. reviewed 10,259 autopsies performed in New York, NY and found the incidence of unruptured cerebral aneurysms highest in patients over 60 years, with a peak prevalence of 1.2% in the seventh decade of life [3]. In the Japanese population, Iwamoto et al. evaluated 1230 autopsies, reporting an overall increase prevalence of unruptured aneurysms with advancing age in women, with a peak prevalence of 14.5% in Japanese women 60–69 years [4]. Population studies using non-invasive imaging show similar results. When reviewing magnetic resonance angiography studies in 8696 asymptomatic Japanese adults, Harada et al. identified unruptured cerebral aneurysms in 3.2%, and the prevalence increased with age in both men and women [5]. Additional studies, however, report only a minimal association between aneurysm prevalence and advanced age. A meta-analysis of 68 studies reporting on 83 study populations and 1450 unruptured cerebral aneurysms in 94,912 patients describes an increase prevalence of unruptured aneurysms with autosomal dominant polycystic kidney disease, family history of aneurysmal subarachnoid haemorrhage and atherosclerosis. However, when compared to patients 80 years and older, there was no difference in aneurysm prevalence by age, except in patients younger than 30 years [6].

### Risk of rupture

The incidence of aneurysm rupture and association with advanced age has been evaluated in multiple prospective studies. In the general population, the overall risk of rupture per patient-year at risk is 0.6–1.3% [7]. The International Study of Unruptured Intracranial Aneurysms (ISUIA) found a size greater than 7 mm and the location at the basilar tip or posterior communicating artery to be significant risk factors for rupture [2]. A meta-analysis of 19 studies with 4705 patients and 6556 unruptured aneurysms reported a size greater than 5 mm, posterior circulation, female gender and symptomatic aneurysms to be significantly associated with rupture [7]. Additional pertinent risk factors include history of subarachnoid haemorrhage, hypertension, smoking, multiple aneurysms and irregular morphology [2,8–11]. Some report that aneurysm size may be related to patient age. The Unruptured Cerebral Aneurysm Study of Japan (UCAS Japan) evaluated 6697 unruptured cerebral aneurysms and found aneurysms 7 mm or larger in 39.7% of patients 80 years and older compared to 21.4% of patients 50–59 years [9]. While elderly individuals may have a higher incidence of larger aneurysms, the association between age and rupture remains unclear due to confounders such as life expectancy.

The overall rupture rate in elderly patients appears similar to the general population. A pooled analysis evaluating 1896 patients 70 years and older with 2227 unruptured aneurysms reported an annual rupture rate of 1.6% [8]. Aneurysm size may be particularly important in determining the rupture risk in elderly patients. Rinaldo et al. followed the clinical course of patients 65 years or older with unruptured cerebral aneurysms for a median of 3.7 years. The annual risk of rupture was 0.9% and all aneurysms that ruptured were at least 10 mm [12]. Additional studies regarding small aneurysms find rupture to be more common in younger patients. Sonobe et al. evaluated the natural history of unruptured aneurysms 5 mm or smaller and reported an annual rupture risk of 0.54%, with age <50 years being a significant predictive factor of rupture [11]. Additional considerations, including location along the internal carotid artery – posterior communicating artery and the Population-Hypertension-Age-Size-Earlier haemorrhage-Site (PHASES) score, remain independent risk factors for rupture in the elderly, similar to the general population [8,12].

## Key challenges in elderly patients

### Frailty

Frailty is defined as a decrease in homeostatic reserve and is closely associated with increasing age [13]. Frailty is characterized by weakness, exhaustion, low activity, slowed performance and weight loss. Frailty results from chronic inflammation involving multiple organ systems, and is due to a combination of advanced age, genetics, disease, lifestyle and environmental influences that ultimately lead to disability, dependency and death. Frailty is associated with increased postoperative complications, mortality, hospitalization, and institutionalization across multiple surgical procedures [14,15]. A frail patient has reduced physiologic reserve and resistance to stressors causing vulnerability to adverse outcomes; therefore, an understanding of baseline frailty levels allows the tailoring of conservative or aggressive aneurysm management to match the physiologic reserve of elderly patients.

Numerous attempts have been made to quantify frailty based on phenotypic characteristics, functional deficits, social situations and medical comorbidities, with the modified frailty index (mFI) representing a well establish metric of physiologic reserve. Including five (mFI-5) or eleven (mFI-11) factors, based on the original 70-item Canada Study of Health and Ageing Frailty Index, the mFI predicts morbidity and mortality across all subspecialties [16]. In neurosurgery, frailty is a predictor of worse outcomes following angiogram-negative subarachnoid haemorrhage [17], brain tumour resection [18] and ischaemic stroke [19].

Brain frailty resulting from intrinsic damage to small perforating arterioles, endothelial dysfunction, blood–brain barrier breakdown and inflammation is an additional marker of poor cerebral reserve in elderly patients, and is readily evident on advanced imaging as white matter hyperintensities, microbleeds, prominent perivascular spaces, lacunar infarcts and cerebral atrophy. Baseline brain frailty scores on magnetic resonance imaging and computed tomography are associated with poor functional outcomes following lacunar and non-lacunar stroke [20]. Brain frailty, along with age, are significant risk factors for surgical clipping of unruptured cerebral aneurysms. Matano et al. evaluated 214 patients with a mean age of 68 years who underwent clipping of unruptured aneurysms; they found that severe baseline white matter lesions were significantly correlated with poor outcome, and were associated with increased risk of ischaemia, haemorrhage, epilepsy and length of hospital stay [21]. Additional studies regarding the association between frailty and outcomes following surgical or endovascular management of unruptured cerebral aneurysms are lacking, but a review of 25 studies published between 2015 and 2018 found frailty associated with worse outcomes across the neurosurgical subspecialties [22]. In practice, frailty has important management implications because physiologic and cerebrovascular reserve influence the risk-benefit ratio of aneurysm treatment. An elderly, frail patient may be better served with a conservative approach or treatments that prioritize reduced procedure time and anaesthesia exposure along with shorter hospital stays. In addition, surgical, endovascular and anaesthetic techniques prioritizing cerebral homeostasis over more aggressive, prolonged or repeated treatments may be considered due to the increased vulnerability to adverse outcomes associated with frailty.

### Cognitive function

Preserved cognitive function is particularly relevant in elderly patients, as any neuropsychological decline either from the anxiety of harbouring an unruptured aneurysm or as a direct consequence of treatment may have dramatic consequences on quality of life, independence and overall life expectancy. Patients with unruptured cerebral aneurysms may harbour cognitive impairments at baseline, which can influence long-term outcomes. Fukunaga et al. evaluated 30 patients with unruptured cerebral aneurysms and described neuropsychological impairment in three [23]. Haug et al. observed baseline mild impairments in executive function, motor speed and visual memory in 15 patients with unruptured middle cerebral artery aneurysms [24]. Cognitive impairment may result from generalized cerebral vasculopathy related to chronic hypertension and tobacco use, which are principal risk factors in both aneurysm development and chronic ischaemic white matter disease. Limited cerebrovascular reserve becomes more pronounced with advanced age and is highly variable, correlating with the heterogeneous cognitive outcomes following unruptured aneurysm treatment.

Current literature is inconclusive regarding cognitive outcomes following elective aneurysm treatment. ISUIA reported impaired cognitive function in 3.2–7.1% of patients who underwent either clipping or coiling at 1 year after treatment [2]. Post-operative cognitive dysfunction may be related to structural damage to the brain parenchyma. Inoue et al. demonstrated a positive correlation between post-operative T2 imaging changes and decreased neurocognitive scores following elective aneurysm clipping [25]. Particularly vulnerable are patients with baseline chronic ischaemic small vessel disease. In a study of 106 patients who underwent clipping of unruptured cerebral aneurysms, Kumon et al. found moderate to severe deep white matter hyperintensities on preoperative MRI independently associated with post-operative neurocognitive impairment [26]. Additional reports describe neurocognitive dysfunction associated with specific surgical approaches, aneurysm locations and age. Ohue et al. reported deteriorating neurocognitive function in 17 of 43 patients with unruptured cerebral aneurysms treated surgically, with greater tendency towards decline found in patients 65 years or older, those with anterior communicating artery aneurysms, and those requiring an interhemispheric approach [27].

Additional studies, however, report no difference in cognitive function before *vs.* after treatment of an unruptured aneurysm. Srivatsan et al. evaluated Montreal Cognitive Assessment scores following elective coiling of unruptured aneurysms in 33 patients and found no difference compared to baseline scores [28]. Similarly, Wagner et al. found no difference between baseline and post-operative neurocognitive function in 51 patients with unruptured cerebral aneurysms treated with flow diversion [29]. Few studies have been completed specifically in the elderly population. Kubo et al. found no impairment in cognitive function and overall decreased anxiety following microsurgical clipping of unruptured aneurysms in patients 70 years and older, with the greatest reductions in anxiety seen in patients with the highest preoperative cognitive function scores [30].

Given the lack of definitive guidelines, conflicting outcomes regarding cognitive function following treatment, and few studies in the elderly, neurocognitive prognosis must be highly individualized. New evaluations in elderly patients should emphasize the anxiety of harbouring an unruptured aneurysm, which may improve with definitive aneurysm repair. Cerebrovascular cognitive reserve, baseline ischaemic white matter disease and evidence of vascular cognitive impairment are particularly important. Meticulous surgical dissection and endovascular technique may minimize parenchymal injury and preserve cognitive function post-operatively. Nevertheless, patients with extensive chronic ischaemic changes may be unfavourable candidates for treatment due to increased susceptibility to long-term neurocognitive changes, as seen in the landmark studies such as ISUIA [2].

### Reduced life expectancy

The long-term risks of unruptured cerebral aneurysms in the elderly are challenging to determine based on the existing literature. As life expectancy declines, the likelihood of rupture combined with comorbidities that are more likely to cause death may call into question the benefit of aneurysm treatment. Vindlacheruvu *et al.* performed a life expectancy analysis of patients with unruptured aneurysms with and without repair based on prospective data from ISUIA to identify circumstances under which aneurysm repair would be beneficial [31]. They found that in patients with aneurysms 7 mm or larger, or with aneurysms of the posterior circulation, life years are gained with repair up to age 45–65 or in those with life expectancies greater than 15–35 years. This leaves uncertain the best course of action for unruptured aneurysms in patients 65 years or older.

Pre-existing medical conditions and comorbidities, along with age, predict remaining life years following hospitalization. One year survival after first admission for heart failure or initiation of dialysis is 67% and 45.5%, respectively [32,33]. Elderly patients have higher prevalence of congestive heart failure, COPD, diabetes mellitus, hypertension, peripheral vascular disease and chronic renal failure, and these comorbidities increase rates of in-hospital mortality following treatment of unruptured cerebral aneurysms. Silva et al. retrospectively reviewed patients 65 years and older with unruptured aneurysms treated with either clipping or coiling [34]. They found that elderly patients treated with microsurgical clipping had higher perioperative mortality rates compared to non-elderly counterparts. Moreover, elderly patients had higher rates of peri-intervention stroke, cardiac dysfunction and acute renal failure following clipping and coiling compared to non-elderly.

### Vasculopathy

Chronic hypertension and atherosclerotic disease, common comorbidities that increase with advancing age, may accelerate vessel or aneurysm calcification, reduce arterial compliance, increase vessel tortuosity and narrow parent vessels, thereby complicating treatment. Atherosclerotic aneurysm calcification is more common in larger aneurysms and in older patients, and is associated with increased morbidity following clipping. In a study of 208 patients treated with clipping or coiling of unruptured aneurysms, the presence of calcification in an aneurysm was the sole marker of adverse outcome [35]. In addition, chronic hypertension can lead to complex aortic arch features and aortic unfolding that, along with carotid artery tortuosity and reduced vessel compliance, complicate arterial navigation, increase technical difficulty, and increase the risk of ischaemic complications in endovascular procedures [36].

Intracranial atherosclerotic disease adjacent to cerebral aneurysms also influences the efficacy and complication profile of endovascular treatment. Gao *et al.* found that arterial stenosis and tortuous anatomy limits the spread of microcatheter tension, may increase the risk of intra-procedural aneurysm rupture and plaque mobilization, decreases the rate of complete occlusion, and may necessitate the use of adjuvant stent or balloon assistance, which increases the risk of in-stent thrombosis and arterial dissection [37]. While endovascular treatment of intracranial aneurysms with adjacent atherosclerotic narrowing is feasible, careful preprocedural assessment of aneurysm morphology, parent artery anatomy and collateral circulation is needed to minimize complications [37].

### Prognosis in case of rupture

Advanced age is recognized as a poor prognostic indicator after subarachnoid haemorrhage, and the overall worse outcome in elderly patients following aneurysm rupture has important implications regarding patient counselling and management. Lanzino et al. evaluated the relationship between age and outcome in aneurysmal subarachnoid haemorrhage using data from the multicentre randomized trial of nicardipine in subarachnoid haemorrhage [38]. They reported that the level of consciousness, the grade of subarachnoid haemorrhage, and adverse radiographic features such as thick clot, hydrocephalus and intraventricular haemorrhage worsened with advancing age. Life-threatening complications also increased, and outcomes, as measured by Glasgow Outcome scores, were worse in elderly compared to younger patients [38]. Additional studies found favourable outcomes infrequent in high grade elderly subarachnoid haemorrhage patients [39,40]. Even elderly patients with good neurological grades may have worse outcomes compared to younger patients. Catapano *et al.* analysed outcomes of elderly patients with aneurysmal subarachnoid haemorrhage stratified by age and clinical presentation [41]. They reported that those 65 years and older were more likely to have poor functional outcomes compared to patients 60–65 years, and among good grade patients, those 65 years and older had poorer outcomes than the 60–65 years cohort at 6-year follow-up.

## Treatment of unruptured cerebral aneurysms in the elderly

### Endovascular coiling

Coiling provides a minimally invasive alternative to open surgery for treatment of intracranial aneurysms. Coiling is generally the favoured treatment in elderly patients despite a lack of supportive randomized controlled trials. For ruptured aneurysms, the landmark International Subarachnoid Aneurysm Trial (ISAT) in 2002 [42] and 2015 [43] shaped the preference for coiling of ruptured cerebral aneurysms, and a subgroup analysis of 278 patients greater than 65 years found that coiling was the favoured treatment for good grade haemorrhages with small anterior circulation aneurysms [44]. However, the generalizability of this practice for unruptured aneurysms remains unclear. With a fundamentally different natural history and higher prevalence of vessel tortuosity, aortic unfolding and intrinsic vessel disease, the risk-benefit ratio of coiling in the elderly remains highly individualized.

Coiling of unruptured aneurysms in the elderly appears overall to be safe and effective. Hwang et al. coiled 122 saccular unruptured aneurysms in 96 consecutive patients 70 years or older; they successfully occluded the aneurysm with no adverse clinical events in 95.9% of cases [45]. A meta-analysis of 21 studies reporting on 1511 patients 65 years or older with ruptured and unruptured aneurysms treated with coiling reported long-term occlusion in 79% [46]. Perioperative stroke, intraprocedural rupture and peri-operative mortality rates were 4%, 1% and 1%, respectively, in patients with unruptured aneurysms, and rates of good clinical outcomes at 1 year were 93% [46]. Additional reports highlight the increased complexity of coiling in elderly patients. In patients 70–96 years, Gonzalez et al. found wide-necked aneurysms more common in patients without subarachnoid haemorrhage [47]. With coiling, complete occlusion was achieved in only 28%, and 11% had unsuccessful coiling due to microcatheter instability associated with tortuous anatomy and wide neck morphology. Despite high rates of incomplete obliteration, rupture was only encountered in one patient during follow-up, and good clinical outcome was achieved in 91% [47]. It remains unclear if complete obliteration is really necessary in elderly patients, or if targeted subtotal occlusion is sufficient, particularly with wide-necked, dysplastic aneurysms that are relatively small.

Elderly patients may be particularly prone to complications following coiling of unruptured aneurysms. Khosla et al. compared the complication profile associated with elective coiling of intracranial aneurysms between elderly and non-elderly patients, reporting major complications with and without neurologic disability more prevalent in patients 65 years and older [48]. Ischaemic complications may be more likely in elderly patients with increased rates of atherosclerosis, vessel rigidity, arterial tortuosity and reduced vascular reserve. Additional efforts to reduce procedural time, avoid neck overpacking, minimize coil prolapse and optimize medical comorbidities should be emphasized.

### Adjuvant endovascular techniques: balloon and stent assistance

Adjuvant techniques for coil embolization, including balloon assistance and stent assistance, have been studied only modestly in the elderly, potentially due to their relatively infrequent usage. In a meta-analysis by Sturiale et al. evaluating 21 studies with 1511 elderly patients undergoing endovascular treatment of both ruptured and unruptured aneurysms, only 5% utilized balloon assistance and 3% utilized stent assistance [46]. Balloon and stent-assistance allow “remodelling” of wide-necked aneurysms and protection against coil prolapse, extending the capabilities of coiling to include aneurysms with unfavourable aspect ratios, branch or parent vessel incorporation and significant dysplasia. The use of adjuvant techniques to achieve occlusion in more complex aneurysms often portends increased risk with increased procedural time and thrombogenicity of stents and balloons. Benitez et al. examined the Neuroform microstent in the treatment of wide-necked cerebral aneurysms and reports death in 8.9% and thromboembolic events in 7% [49]. Additional reports of Neuroform microstent-assistance report thromboembolic complications in 11% [50]. Elderly patients may be particularly susceptible to the increased complication profile of adjuvant endovascular techniques. Stiefel et al. reported a single-centre experience with endovascular treatment of unruptured aneurysms in 77 patients 70 years or older, with adjuvant stent or balloon assistance used in 66% of cases [51]. Stand-alone coiling was significantly safer compared to balloon or stent assistance, with a complication rate of 6% compared to 27% and 26% for stent and balloon assistance, respectively. Similar to the general population,11% of adjuvant cases suffered thromboembolic complications, and permanent morbidity and mortality were observed in 5% [51]. When considering more aggressive endovascular interventions, the increased complication profile of adjuvant endovascular techniques requires careful evaluation of aneurysm risk profile and patient physiological reserve.

### Microsurgical clipping

Microsurgical clipping of unruptured aneurysms is a more invasive alternative to endovascular treatment, and elderly patients are less likely to tolerate clipping as compared to younger patients. Barker et al. compared age-dependent outcomes in 3498 patients who underwent clipping or coiling for unruptured aneurysms [52]. When discharge to short-term rehabilitation was counted as an adverse event, coiled patients had significantly better outcomes than clipped patients at the time of hospital discharge, and most of the coiling advantage was concentrated in patients older than 65 years [52]. Brinjikji et al. reviewed a national inpatient registry of 63,940 patients with unruptured cerebral aneurysms treated with clipping or coiling to determine the effect of age on outcomes [53]. They found that improvements in outcome with coiling relative to clipping are amplified in elderly patients. Patients 65 years and older who underwent clipping had a mortality rate of 2.5% compared to only 0.9% for patients undergoing coiling. The difference in discharge to long-term facility rate was also marked, occurring in 27% of patients undergoing clipping compared to only 7.4% for patients treated with coiling [53]. The trend towards worse outcomes with microsurgical clipping in elderly patients is also reported in a subgroup analysis ISUIA. Mahaney et al. found that while surgical treatment of unruptured aneurysms prevents 1-year haemorrhage and confers a survival benefit in patients 50 years or younger, significant risk of morbidity and death is observed in patients 65 years or older [54]. Additional studies report clipping associated with higher rates of immediate post-procedure complications compared to coiling in elderly patients [55].

Increased morbidity, mortality and institutionalization following discharge for microsurgical clipping of unruptured aneurysms in elderly patients suggests the need for less invasive surgical techniques focussed on minimal tissue disruption. The superciliary keyhole approach has been advocated as an alternative to pterional craniotomy for anterior circulation aneurysms, but validation in elderly patients has not been described [56]. A retrospective study by Mori et al. comparing the safety and efficacy of keyhole clipping *via* a supraorbital or pterional approach in 62 non-frail elderly and 198 non-elderly patients reported no increase in complication rates, hospital duration, aneurysm recurrence, morbidity or mortality with advanced age [57]. A minimal craniotomy *via* a supraorbital or pterional approach, sufficient drilling of the frontal skull base or sphenoid ridge, and wider subdural spaces in elderly patients improved aneurysm access and minimized excessive brain retraction, contributing to lower rates of neurological morbidity, mortality and cognitive dysfunction compared to standard clipping.

### Flow diversion

Flow diverting stents are approved for the treatment of cerebral aneurysms of the internal carotid artery. Their reduced porosity creates a scaffolding for endothelialization across the cerebral aneurysm neck, reducing intra-luminal flow and allowing for slow obliteration. No recurrences have been reported once occlusion is achieved, and flow diversion has become a standard treatment for wide-necked saccular or fusiform internal carotid artery aneurysms for which traditional endovascular and surgical treatments are limited.

Elderly patients have reduced but acceptable rates of aneurysm occlusion following flow diversion. Kuhn et al. evaluated the effects of age on aneurysm occlusion following flow diversion for treatment of unruptured cerebral aneurysms [58]. At 12–18 months follow-up, near complete to complete occlusion was 100% in patients less than 45 years, 91.4% in patients 46–64 years, and 78.4% in patients 65 years and older. Adeeb et al. reported similar results, finding that age 70 years and older and non-smoking status were independent risk factors for incomplete aneurysm occlusion [59]. Despite these limitations, the occlusion rates for challenging small, medium and large wide-neck aneurysms remain impressively high, and flow diversion remains a valid treatment in properly selected elderly patients.

Older patients treated with flow diversion are more likely to harbour aneurysms of larger size, complex morphology and within the posterior circulation compared to younger counterparts which, along with increased frequency of comorbidities, atherosclerosis and arterial tortuosity, raises concern for increased morbidity and mortality. Brinjikji et al. reviewed age-related outcomes following pipeline reconstruction of unruptured aneurysms in 711 patients and found increasing age associated with higher neurological mortality [60]. Patients 70 years and older had neurological mortality rates of 7.4% compared to 3.3% for patients 61–70 years, 2.7% for patients 51–60 years and 0.5% for patients 50 years and younger. Similar rates of intracerebral haemorrhage and ischaemic stroke were seen across age groups, suggesting elderly patients suffer more morbidity and mortality from a given ischaemic or haemorrhagic insult. Meticulous technique, standardized antiplatelet regiments and careful patient selection should be emphasized to keep complications of flow diversion acceptably low; age alone should not be an exclusion criterion.

### Medical therapy

#### Aspirin

Aspirin lowers rates of aneurysm rupture in animal models *via* inhibition of cyclooxygenase-2 (COX-2), supporting the inflammatory hypothesis of aneurysm formation and rupture [61]. In a small pilot study, biopsy of aneurysm domes taken during elective clipping of patients on aspirin found lower expression of COX-2 compared to those not taking aspirin [62].

Preliminary prospective and retrospective studies suggest that aspirin is safe in patients with unruptured aneurysms and may lower the risk of aneurysmal subarachnoid haemorrhage. A prospective, multicentre observational study that followed patients with unruptured cerebral aneurysms and ischaemic cerebrovascular disease found that aspirin was safe in patients with concurrent small unruptured cerebral aneurysms and was associated with low rate of aneurysm rupture [63]. Hasan et al. performed a subgroup analysis of ISUIA and found patients who used aspirin three times weekly to daily had significantly lower odds of haemorrhage compared with those never on aspirin [64]. Similarly, a study of 1340 patients with aneurysmal subarachnoid haemorrhage compared to 10,000 matched controls found aspirin use, especially for three or more years, associated with decreased risk of subarachnoid haemorrhage [65].

Aspirin may also limit aneurysm growth. A prospective cohort study following 272 patients with unruptured aneurysms 7 mm or smaller, 113 of which were on aspirin continuously, found that aspirin was associated with a low risk of aneurysm growth [66]. An additional retrospective study evaluated the protective effects of aspirin on aneurysm growth in patients harbouring multiple intracranial aneurysms 5 mm or smaller [67]. Aspirin was significantly associated with decreased rate of growth while hypertension, drug abuse, polycystic kidney disease and prior subarachnoid haemorrhage were associated with increased rate of growth. While aspirin may be associated with reduced aneurysm growth and rupture, in healthy older adults, aspirin has been associated with increased all-cause and cancer-related mortality [68]. Therefore, the protective benefits of aspirin need to be balanced with the overall increase in mortality from additional causes.

#### Statins

Statins have anti-inflammatory effects in various vascular diseases, but their protective effects on the formation and progression of cerebral aneurysms remain unclear. A multicentre case–control study compared 117 patients with aneurysmal subarachnoid haemorrhage to 304 patients with incidental, unruptured cerebral aneurysms and found a lower rate of statin use in rupture cases, suggesting an inverse relationship between statin use and aneurysm rupture [69]. Alternatively, a retrospective cohort study examining 28,931 patients with unruptured cerebral aneurysms found no significant effect of statin use on likelihood of aneurysm rupture [70]. Currently, the Statin Treatment for UnruptureD Intracranial anEurysms (STUDIES) trial, a randomized, placebo-controlled trial to assess whether atorvastatin leads to significant changes in aneurysm vessel wall enhancement and inflammatory markers is ongoing [71].

## Future directions

Future alternatives to stratify treatment decisions in high-risk elderly patients, evaluation of new endovascular devices and microsurgical innovation will continue to improve elderly patient outcomes. Wall enhancement on black-blood MRA, observed more frequently in unstable, growing and ruptured aneurysms [72,73], may improve our understanding of aneurysm-specific rupture risk in the elderly, and help guide treatment. Validation of intra-saccular flow diverters and the capacity for endothelization following Woven EndoBridge (WEB) deployment need to be established in elderly patients. Continued advancement of minimally invasive surgical approaches prioritising reduced tissue injury, endoscopic assistance to improve visualization and transnasal approaches for high-risk skull base lesions may be particularly applicable in elderly patients at higher risk for more traditional surgical strategies. Overall, as technological innovations provide more minimally invasive treatments, validation and application in the elderly will be particularly important.

## Conclusions

The optimum management of unruptured cerebral aneurysms in elderly patients remains undefined. The process of ageing presents unique considerations and challenges, and management is currently individualized, requiring precise knowledge of natural history and risk of rupture that must be weighed against an individual’s comorbidities, life expectancy, treatment-related risks and likelihood of treatment success. This report provides a comprehensive review of unruptured cerebral aneurysms in the elderly, with a special focus on the key challenges that this age group presents, including frailty, cognitive dysfunction, reduced life expectancy, vasculopathy and poor prognosis in case of rupture. These challenges determine the risk, benefit and likelihood of success of both surgical and endovascular treatments, drive innovative surgical techniques, and contribute to the trend in endovascular treatment in patients of advanced age. While coiling with or without adjunctive techniques or flow diversion will continue to increase, robust preclinical studies regarding the role in neuroinflammation in aneurysm development, growth and rupture represent potential future targets that may be particularly relevant to the elderly population. We anticipate that this review will provide a clinical framework for managing unruptured aneurysms in this challenging cohort, which will only enlarge with time as the population ages.

## Data Availability

Data sharing is not applicable to this article as no new data were created or analysed in this study.

## Abbreviations

ISUIA: International Study of Unruptured Intracranial Aneurysms
UCAS: Unruptured Cerebral Aneurysm Study
PHASES: Population Hypertension Age Size Earlier haemorrhage Site score
mFI: modified frailty index
ISAT: International Subarachnoid Aneurysm Trial
COX-2: Cyclooxygenase-2
MRI: Magnetic Resonance Imaging
WEB: Woven EndoBridge
MRA: Magnetic Resonance Angiography
